# Commentary: “Healthcare Professionals’ Preferred Efficacy Endpoints and Minimal Clinically Important Differences in the Assessment of New Medicines for Chronic Obstructive Pulmonary Disease” by Dankers M et al. in *Frontiers in Pharmacology* 2020; 10: 1519

**DOI:** 10.3389/fphar.2020.00827

**Published:** 2020-06-02

**Authors:** Harma Alma, Corina de Jong, Janwillem Kocks, Thys van der Molen

**Affiliations:** ^1^Department of General Practice and Elderly Care Medicine, University of Groningen, University Medical Center Groningen, Groningen, Netherlands; ^2^GRIAC Research Institute, University of Groningen, University Medical Center Groningen, Groningen, Netherlands; ^3^General Practitioners Research Institute, Groningen, Netherlands; ^4^Observational and Pragmatic Research Institute, Singapore, Singapore

**Keywords:** health status, COPD, (MCID) minimal clinically important difference, St. George’s Respiratory Questionnaire, patient-reported outcomes

## Introduction

Pharmacological clinical trials require obligatory endpoints in evaluating the clinical relevance of their outcomes (Jones, 2001; US Department of Health and Human Services FDA Center for Drug Evaluation and Research, 2006). The minimal clinically important difference (MCID) is an important parameter that represents the threshold of an outcome measure at which the observed change can be considered clinically relevant for the patient, hence justifying the therapy (Jaeschke et al., 1989). Frequently applied endpoints in clinical trials for patients with chronic obstructive pulmonary disease (COPD) include spirometry with forced expiratory volume in one second (FEV_1_), exacerbations, and health status (US Department of Health and Human Services FDA Center for Drug Evaluation and Research, 2006; Jones et al., 2012; Cazzola et al., 2015).

The most frequently selected health status instrument in scientific research is the St. George’s Respiratory Questionnaire (SGRQ) with a proclaimed MCID of four units on a total scale from 0 to 100 (Jones et al., 1991; US Department of Health and Human Services FDA Center for Drug Evaluation and Research, 2006; Jones et al., 2012). Higher scores represent worse health status. MCIDs can be determined using anchor-, distribution-, or opinion-based methods (Copay et al., 2007). It is with great interest that Dankers et al. have investigated healthcare professionals’ opinion on among others the MCID threshold of the SGRQ. To our knowledge, this is the first study to investigate these expert-ratings on the MCID of the SGRQ. The authors’ conclusion was that healthcare professionals prefer a higher cutoff value for clinical relevance of 11 on the SGRQ instead of the currently used MCID of four by registration authorities.

## Redefining the MCID of the SGRQ

We agree with Dankers et al. that the MCID of the SGRQ should in fact be higher than four points, and that current clinical trials hence may have overestimated the interpretation of treatment effects. In general, MCIDs of instruments are approximately 7–10% of the maximum scale score (Copay et al., 2007). This would represent a change of 7–10 points on the SGRQ. In a published systematic review in the *European Respiratory Journal*, Alma et al. (2018b) reviewed the available content on the MCID of COPD health status tools including the SGRQ (Alma et al., 2018b). A meta-analysis by means of triangulation procedures was performed and resulted in weighted MCID cutoff values of −7.43 (4 studies, range −10.19 to −2.40) for the SGRQ; −2.54 (6 studies, range −3.80 to −1.00) for the COPD Assessment Test (CAT); and −0.43 (5 studies, range −0.62 to −0.21) for the Clinical COPD Questionnaire (CCQ).

**Figure 1:**
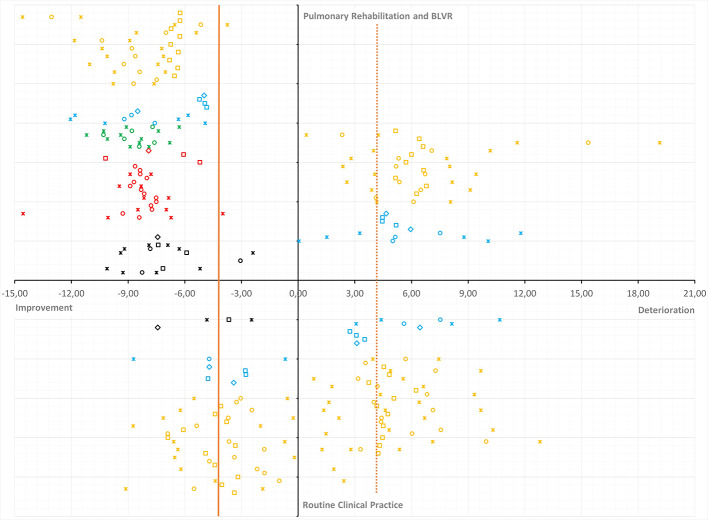
Plot of minimal clinically important difference (MCID) estimates for the St. George’s Respiratory Questionnaire (SGRQ) from recent publications and systematic review. Overview of MCID estimates for improvement anddeterioration (horizontal axis) from intervention/PR versus RCP (vertical axis). Estimates are presented as circles (anchor-based estimates), squares (distribution-based estimates), diamonds (weighted or triangulated MCIDs), or asterisks (lower and upper limit of the observed range or 95% CI). The vertical orange reference line represented the currently accepted MCID in the literature. The dashed vertical orange reference line mirrors the accepted MCID for improvement into deterioration. MCID estimates are color coded per publication: systematic review and triangulation (black) (Alma et al., 2018b); various MCID methods (red) (Alma et al., 2016); follow-up period and anchor question (green) (Alma et al., 2018a); improvement versus deterioration (blue) (Alma H.J. et al., 2019); MCID and dynamic factors (orange) (Alma H. et al., 2019).

Moreover, multiple publications emerged over the past few years on the MCID of the SGRQ, proclaiming that this threshold should in fact be higher than four units (Welling et al., 2015; Alma et al., 2016; Alma et al., 2018a; Alma H.J. et al., 2019; Alma H. et al., 2019). **Figure 1** summarizes the evidence from these publications. The majority of estimates is in the range of five to nine points depending on the study setting as well as the direction of the measured change (*improvement versus deterioration*). Only MCID estimates from routine clinical practice come somewhat close (range three to six points) to the proclaimed MCID of four units (Jones, 2005).

We would like to highlight that experts’ opinions on the MCID of the SGRQ are of interest as they convey a message of clinical judgment; however there is clear evidence that ratings by physicians and patients on quality of life and MCIDs are not in line (Janse et al., 2004). What relevance does an expert-based MCID of 11 units have, if it does not match with the experienced change by the patient with COPD? Especially since the SGRQ is defined as a patient-reported outcome (PRO). It is also remarkable that experts in the Netherlands were asked about a questionnaire like the SGRQ, without being very familiar with it. Ratings were based upon a short description of the questionnaire and its total scoring range. On what was the respondent’s judgment based? Was it just a gut-feeling or was there actually rationale for? Since it is known that Dutch healthcare professionals have more experience with the CCQ (Van der Molen et al., 2003; Snoeck-Stroband et al., 2015), one would think ratings for this clinically applied tool, would be more reliable than for an instrument that is infrequently used. It would make more sense to formulate a threshold for something complicated as an MCID, once the professional has knowledge about its content and clinical application.

It is also interesting to have a closer look at the number of responders in this analysis, since this was less than 3% of the group of invited professionals (227 replied out of 7,731 invitations). Is this representative and was this in fact the group of professionals most frequently working with patients with COPD and the SGRQ? Especially since approximately half of this group was a pharmacist, rather than a physician or practice nurse. Although pharmacists play a key role in the support of patients with COPD, they do not work directly with health status instruments in their day-to-day practice. This inexperience may also be reflected in the large variation of responses by the healthcare professionals, because the standard deviation was 10.1 out of the rated overall MCID of the SGRQ of 11 units.

Last, but not least it is unknown if the professionals reviewed thresholds at the individual level or at the group level (Beaton et al., 2001; Kocks et al., 2010). Physicians and healthcare professionals meet the individual patient in clinical practice, possibly requiring more change after intervention. In scientific research, conclusions are drawn based on reaching the MCID threshold at the group level. Extreme high and low change scores are usually integrated in the regression to the mean phenomenon.

## Discussion

Dankers et al. have an important share in continuing the discussion on the MCID of the SGRQ. Experts’ opinions on the threshold of the SGRQ could be valuable, yet should not be leading. It is very remarkable that despite new studies on the MCID of the SGRQ, the threshold of four points (Jones, 2005)—based upon unclear methodology—is still applied in scientific research. If we continue doing so, trial outcomes could be severely overestimated in its interpretation. Costly therapies may be offered to patients without a valid basis. Possibly with some good individual responses, but at the group level they may not meet sufficiently clinically relevant improvements. The newer health status instruments (like the CCQ and CAT) have shown better effect sizes and responsiveness, and their MCID is more rigorously determined. Integrating these measures in future studies may lead to better assessment of treatment response than continuing using the SGRQ.

## Author Contributions

HA wrote the first draft. CJ, JK, and TM reviewed and finalized the manuscript.

## Funding

Junior Scientific Masterclass of the University of Groningen.

## Conflict of Interest

TM holds the copyright of the Clinical COPD Questionnaire.

The remaining authors declare that the research was conducted in the absence of any commercial or financial relationships that could be construed as a potential conflict of interest.

## Abbreviations

95%CI, 95% confidence interval; BLVR, bronchoscopic lung volume reduction; MCID, minimal clinically important difference; PR, pulmonary rehabilitation; RCP, routine clinical practice; SGRQ, St. George’s Respiratory Questionnaire.
